# Brain atrophy patterns in multiple sclerosis patients treated with natalizumab and its clinical correlates

**DOI:** 10.1002/brb3.2573

**Published:** 2022-04-10

**Authors:** Arwa Rekik, Mona Aissi, Islem Rekik, Mariem Mhiri, Mahbouba Ayed Frih

**Affiliations:** ^1^ Department of Neurology University Hospital Fattouma Bourguiba Monastir Monastir Tunisia; ^2^ Faculty of Medicine of Monastir Fattouma Bourguiba Monastir Tunisia; ^3^ BASIRA Lab Faculty of Computer and Informatics Istanbul Technical University Istanbul Turkey; ^4^ School of Science and Engineering Computing University of Dundee Dundee UK

**Keywords:** atrophy, cognition, multiple sclerosis, natalizumab, progression, segmentation

## Abstract

**Background:**

Multiple sclerosis (MS) is defined as a demyelinating disorder of the central nervous system, witnessing over the past years a remarkable progress in the therapeutic approaches of the inflammatory process. Yet, the ongoing neurodegenerative process is still ambiguous, under‐assessed, and probably under‐treated. Atrophy and cognitive dysfunction represent the radiological and clinical correlates of such process. In this study, we evaluated the effect of one specific MS treatment, which is natalizumab (NTZ), on brain atrophy evolution in different anatomical regions and its correlation with the cognitive profile and the physical disability.

**Methods:**

We recruited 20 patients diagnosed with relapsing‐remitting MS (RR‐MS) and treated with NTZ. We tracked brain atrophy in different anatomical structures using MRI scans processed with an automated image segmentation technique. We also assessed the progression of physical disability and the cognitive function and its link with the progression of atrophy.

**Results:**

During the first 2 years of treatment, a significant volume loss was noted within the corpus callosum and the cerebellum gray matter (GM). The annual atrophy rate of the cortical GM, the cerebellum GM, the thalamus, the amygdala, the globus pallidus, and the hippocampus correlated with greater memory impairment. As for the third and fourth years of treatment, a significant atrophy revolved around the gray matter, mainly the cortical one. We also noted an increase of the thalamus volume.

**Conclusion:**

Atrophy in RR‐MS patients treated with NTZ is regional and targeting highly cognitive regions mainly of the subcortical gray matter and the cerebellum. The cerebellum atrophy was a marker of physical disability progression. NTZ did not accelerate the atrophy process in MS and may play a neuroprotective role by increasing the thalamus volume.

## Graphical abstract

This study demonstrated the regional and dynamic aspect of atrophy progression in multiple sclerosis patients treated with natalizumab and its correlations with both physical disability and cognitive impairment. It also highlighted the effect of such treatment of the thalamus, suggesting a potential neuroprotective role.

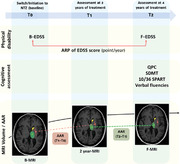

## 1 INTRODUCTION

Multiple sclerosis (MS) is an inflammatory disease of the central nervous system (CNS) involving a complex combination of both demyelination and neurodegeneration (Kotelnikova et al., 2017). The most common course of the disease is relapsing‐remitting MS (RR‐MS), which represents 85% of MS forms (Lublin et al., 2014).

As for the clinical expression, cognitive dysfunction is a prominent feature of MS, occurring even in early stages of the disease (Oset et al., 2020) and has been reported during the pre‐symptomatic phases as a potential revealing sign of the radiologically isolated syndrome (RIS) (Menascu et al., 2019). The radiological correlate of cognitive dysfunction is generally perceived through the atrophy pattern and its severity. Recent studies focused on gray matter (GM) analysis in MS, subcortical deep GM in particular, and demonstrated that it was correlated with cognitive dysfunction in MS patients, noticeable even in the earliest stages of the disease yet without consideration of the potential effect of the ongoing treatment (Eshaghi et al., 2018; Gilmore et al., 2009; Prins et al., 2015). The potential contributing role of treatment adds another layer of complexity to interpret factors leading to cognitive impairment and atrophy progression in MS (Sotirchos et al., 2020). Even though such therapies are known to be efficient to control the disease's activity and disability's progression, its impact on the neurodegenerative process and its clinical expression in terms of cognition are mostly lacking (Compston & Coles, 2002).

Natalizumab (NTZ) is one among the therapies of RR‐MS indicated mainly in forms presenting with high disease activity or aggressive evolution. Its efficacy is established in terms of reducing inflammation by preventing leucocytes from reaching the CNS (Polman et al., 2006). However, the effects of such an aggressive treatment on the neurodegenerative process in MS and its consequences upon the cognitive functions remain controversial (Alvarez et al., 2021; Preziosa, Rocca, Riccitelli, et al., 2020; Talmage et al., 2017). Studies examining atrophy evolution in MS patients treated with NTZ attributed it to the pseudoatrophy phenomenon due to the regression of inflammation in the white matter during the first year of treatment and was as a consequence considered as a further sign of treatment efficacy.

Nevertheless, these previous studies focused mainly on the white matter and specific structures such as the corpus callosum by measuring its index as a representative marker of atrophy in MS patients (Arpín et al., 2016). Lesion burden progression was also a subject of interest and showed no significant correlations with atrophy evolution and cognitive impairment in MS patients (Preziosa, Rocca, Riccitelli, et al., 2020). As for GM, findings about the effect of NTZ remain dispersed (Ciampi et al., 2017; Preziosa, Rocca, Pagani, et al., 2020; Preziosa, Rocca, Riccitelli, et al., 2020).

Thus, we chose to shed light on the effect of NTZ on the brain atrophy progression in RR‐MS patients while focusing on both WM and GM structures and its correlates in terms of disability progression and cognitive impairment. We also aimed to investigate whether atrophy is a regional phenomenon that may potentially be considered as a biomarker of the disease progression.

## 2 METHODS

### 2.1 Study design and participants

#### 2.1.1 Participants

We conducted an observational longitudinal study in the department of neurology of Fattouma Bourguiba Hospital in Monastir Tunisia from 2015 to 2020. We included patients diagnosed with RR‐MS according to McDonald criteria 2017 and who were switched to NTZ during the period of study.

Inclusion criteria were as follows:

– Diagnosis of RR‐MS based on the 2017 Revised McDonald criteria (Thompson et al., 2018),
– Undergoing treatment with NTZ continuously for a 1‐year‐period at least, and
– No other comorbidities that may interfere with cognitive function/brain atrophy progression (epilepsy, brain tumor, stroke, etc.).

Exclusion criteria were as follows:

– Relapse or steroids and/or plasmapheresis treatment within a year before neuropsychological assessment, and
– Existence of neurological signs that could interfere with cognitive evaluation (e.g., significant upper limb impairment, severe ataxia, or optic neuritis).
– The use of cognition‐influencing medication prescriptions (e.g., antidepressants, neuroleptics or anticholinergic drugs) for at least 3 months before the neuropsychological assessment.

Demographic and clinical data

Based on the patients’ medical files, we noted:

– Gender, current age, age at disease onset, diagnosis delay, and disease duration,
– Educational level,
– Type of first‐line therapy, its duration, and reasons leading to treatment switch.

Detailed baseline disability according to the Expanded Disability Status Scale (B‐EDSS) defined as the EDSS score at the initiation of NTZ. Final EDSS (F‐EDSS) was assessed simultaneously when the patient underwent his last neurological examination along with the final MRI scan acquired in 2020. The different functions of the EDSS score were noted: pyramidal, cerebellar, brainstem, sensory, optic, cognitive functions, and the ambulation score,

– Annual rate of progression (ARP) of EDSS score defined as follows:
  ○
$$\begin{array}{c} \mathrm{ARP}\;\left(\mathrm{points}/\mathrm{years}\right)=\frac{\left[F-\mathrm{EDSS}-B-\mathrm{EDSS}]\;(points\right)}{\mathrm{Time}\;\mathrm{interval}\;\mathrm{separating}\;\mathrm{the}\;\mathrm{assessment}\;\mathrm{of}\;\mathrm{both}\;\mathrm{EDSS}\;\mathrm{scores}\;(years)} \end{array}$$
– Annual relapse rate (ARR) (number of relapses/year) before and after initiating of NTZ, and
– Year of switch to NTZ and the treatment's duration by the end of the study period.

#### 2.1.2 Cognitive evaluation

During the last administration of NTZ in hospital, patients underwent, after clear oral consent, a cognitive evaluation along with a neurological examination including the F‐EDSS assessment. We used a brief, applicable and reliable battery of tests assessing the commonly altered cognitive domains in MS:

– *Cognitive complaint questionnaire (QPC)*: All participants were administered the original French 10‐item yes/no questionnaire (Masson, n.d.) assessing the presence of cognitive difficulties in the last six months. A score ≥ 3 pointed to the presence of considerable cognitive complaint.
– Symbol digit modalities Test (SDMT): The test evaluates the visual processing speed or efficiency. It is the most accurate a sensitive test for information processing speed in MS patients in particular (Benedict et al., 2017; Kalb et al., 2018).
– *Verbal fluency tests*: A phonological fluency test and a semantic fluency test (“animals”) were used. The result consisted in the number of correct words cited during the first minute. For both fluency tests, repetitions were not taken into account (Barois et al., 2020). Verbal fluency tasks in MS serve for screening purposes and the detection of executive dysfunction (Delgado‐Álvarez et al., 2021).
– *10/36 Spatial recall test (SPART)*: The test assesses learning capacity and long‐term visuospatial retention. It has been shown to be the most sensitive measures for detecting memory impairment in patients with MS (Dent & Lincoln, 2000; Gerstenecker et al., 2016).

#### 2.1.3 MRI

All patients were scanned using the same MRI system operating at 1.5 Tesla (Philips, Ingenia) within the MRI unit of Fattouma Bourguiba hospital. An MS‐standardized protocol was used which included 5‐mm slices obtained in axial T2, axial FLAIR, sagittal T1, sagittal FLAIR, and axial T1 before and after administration of gadolinium. T1‐weighted sequence was used as an input for the image‐processing pipeline.

Baseline‐MRI (B‐MRI) scan was available for all patients at the initiation of treatment with NTZ. Being part of the routine control, each patient underwent one MRI control per year in order to rule out a progressive multifocal leukoencephalopathy and the occurrence of new active lesions. We compared the baseline scan with the MRI scans at two different timepoints (at 2 years and 4 years of treatment) in order to ascertain the development of atrophy.

#### 2.1.4 Brain segmentation

Segmentation of brain structures based on each subject T1‐weighted MRI was performed automatically using automated *recon‐all* FreeSurfer processing pipeline (version 5.3.0; http://surfer.nmr.mgh.harvard.edu) to obtain the cortical surface reconstruction and tissue‐class segmentation boundaries. No manual editing was performed to keep methods as automated as possible, and scans with segmentation errors/failures were excluded (Yaakub et al., 2020). The quality of brain segmentation was assessed by a neuroimage data processing expert and then by two neurologists trained to interpret MRI scans of inflammatory diseases of the CNS for further quality validation. A total of 34 regions per hemisphere were segmented. We extracted the volume of the following anatomical regions: intracranial (IC) (excluding the volume of ventricles), the total, sub‐cortical and cortical GM, the total white matter (WM), the hippocampus, the corpus callosum (CC), and the amygdala along with different sub‐cortical GM regions (thalamus [T], putamen [PT], globus pallidus [GP], and caudate nucleus [CN]). For bilateral structures, the sums of the right and left volume fractions were used for analysis. For anatomical and subcortical tissue region labeling, a fully automated processing pipeline (FreeSurfer) is deployed as detailed in Fischl et al. (2002, 2004). FreeSurfer first affinely registers each T1‐weighted MRI to a shared common space using MNI305 (Collins et al., 1994) atlas. Next, the variation in the white matter intensity is quantified to remove the B1 bias field estimation. A skull stripped algorithm is then applied using a deformable template model (Ségonne et al., 2004). Following this nonlinear volumetric the MNI305 atlas, a simple label propagation algorithm is used to propagate the labels of the image template in the common atlas to the target‐registered T1‐weighted image (Fischl et al., 2002, 2004). The FreeSurfer‐generated volumes are then measured following this step.

**FIGURE 1:**
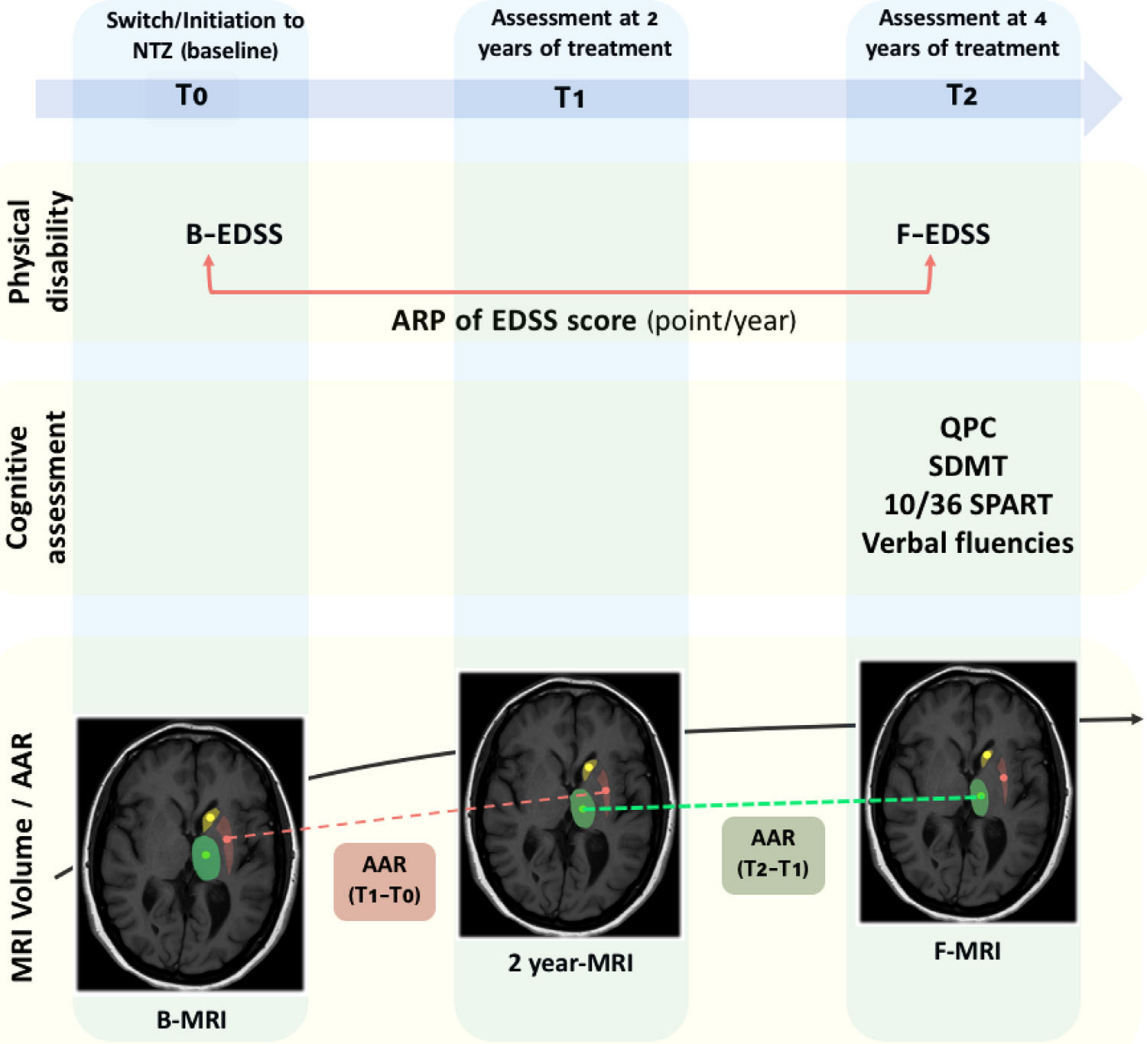
Study design

Assessment of the progression of brain atrophy was based on the comparison of volumes of different structures at different timepoints of the treatment period with NTZ (at 2 years and 4 years of treatment) (Figure 1).

For each anatomical structure, we computed the annual atrophy rate (AAR) using two consecutive timepoints during NTZ treatment period to investigate the progression of atrophy over time (Figure 1).

We defined it as follows: $\mathrm{AAR}\;\left(\mathrm{of}\;\mathrm{the}\;\mathrm{structure}\right)=\frac{\left[\mathrm{Volume}\;\mathrm{of}\;\mathrm{the}\;\mathrm{structure}\;\mathrm{at}\;T(y)-\;\mathrm{Volume}\;\mathrm{of}\;\mathrm{the}\;\mathrm{structure}\;\mathrm{at}\;T(x)\right]}{\mathrm{Time}\;\mathrm{interval}\;\mathrm{separating}\;\mathrm{the}\;\mathrm{two}\;\mathrm{MRI}\;\mathrm{scans}\;(y-x)}\;$where x and y denote the MR scan acquisition timepoints, respectively.

#### 2.1.5 Statistical analysis

For categorical variables, we calculated frequencies. As for quantitative variables, we determined mean score (median if variable was not normally distributed), standard deviation (interquartile deviation if variable was not normally distributed), and minimum and maximum of each variable. Statistical analyses were performed using SPSS (version 22; Chicago, IL, USA). When categorical variables were normally distributed, we used chi‐square test to compare percentages. When the size of the population of study was less than 5, we used Fisher's Test. For comparison between categorical and quantitative variables, we used one‐way analysis of variance (ANOVA).

Pearson's correlation coefficient was used to compare the mean values of two quantitative variables (Mann‐Whitney test if variables were not normally distributed). For parameters that were not normally distributed, non‐parametric approaches were used (Kruskal–Wallis test). The Pearson correlation coefficient, *r*, was indicative of the degree of correlation: 0 indicated a positive association; that is, as the value of one variable increases, so does the value of the other variable. A *p*‐value less than .05 was considered statistically significant in all of the different adopted statistical tests.

#### 2.1.6 Ethics

Since the treatment and follow‐up were managed according to usual clinical practice, ethics committee approval was not required. Nevertheless, informed oral consent was obtained from all participants to undergo the cognitive evaluation.

## 3 RESULTS

### 3.1 Population of study

Twenty patients were finally included in our study among the 25 eligible MS‐patients undergoing NTZ treatment. We excluded one patient who was epileptic with history of two status epilepticus and four patients with distorted segmentation results in order to avoid errors in volume estimation.

### 3.2 Characteristics of the population of study at baseline

Baseline findings are summarized in Table 1.

**TABLE 1:** Baseline characteristics in patients at the initiation of NTZ treatment

Baseline characteristics	Results	
Sex ratio (male:female)		0.66 (8:12)
Educational level	Primary	3
	Secondary	5
	Superior	12
Current age (years) [min; max]		34 ± 7.5 [20; 45]
Mean age of onset (years) [min; max]		26 ± 6 [17; 35]
Mean disease's duration (years) [min; max]		8 ± 5.5 [2; 23]
Type of the first relapse	Motor	11
	Visual	2
	Cerebellar	2
	Brainstem	5
Diagnosis's delay (years)		1 ± 1.8 [0; 6]
First‐line disease‐modifying therapy	Beta‐1b interferon (Betaferon)	8
	Beta‐1a interferon (Rebif)	3
	Beta‐1a interferon (Avonex)	8
Induction therapy		1
Reasons to switch to second‐line therapy	Poor adherence to treatment	6
	Mean AAR [min; max]	1.6 ± 1 [0; 4]
	Increase of number of lesions in MRI control	6
	Presence of active lesions in MRI control	4
	Side effects of treatment	2
Mean baseline‐EDSS [min; max]		3.5 ± 2.5 [1; 6]

*Abbreviations*: AAR, annual atrophy rate; EDSS, Expanded Disability Status Scale; MRI, magnetic resonance imaging.

One patient was treatment‐naïve when NTZ was initiated. The choice of induction therapy was based on his age at disease onset (<18 years old) and the aggressive form of the disease (>2 relapses during the first year).

**FIGURE 2:**
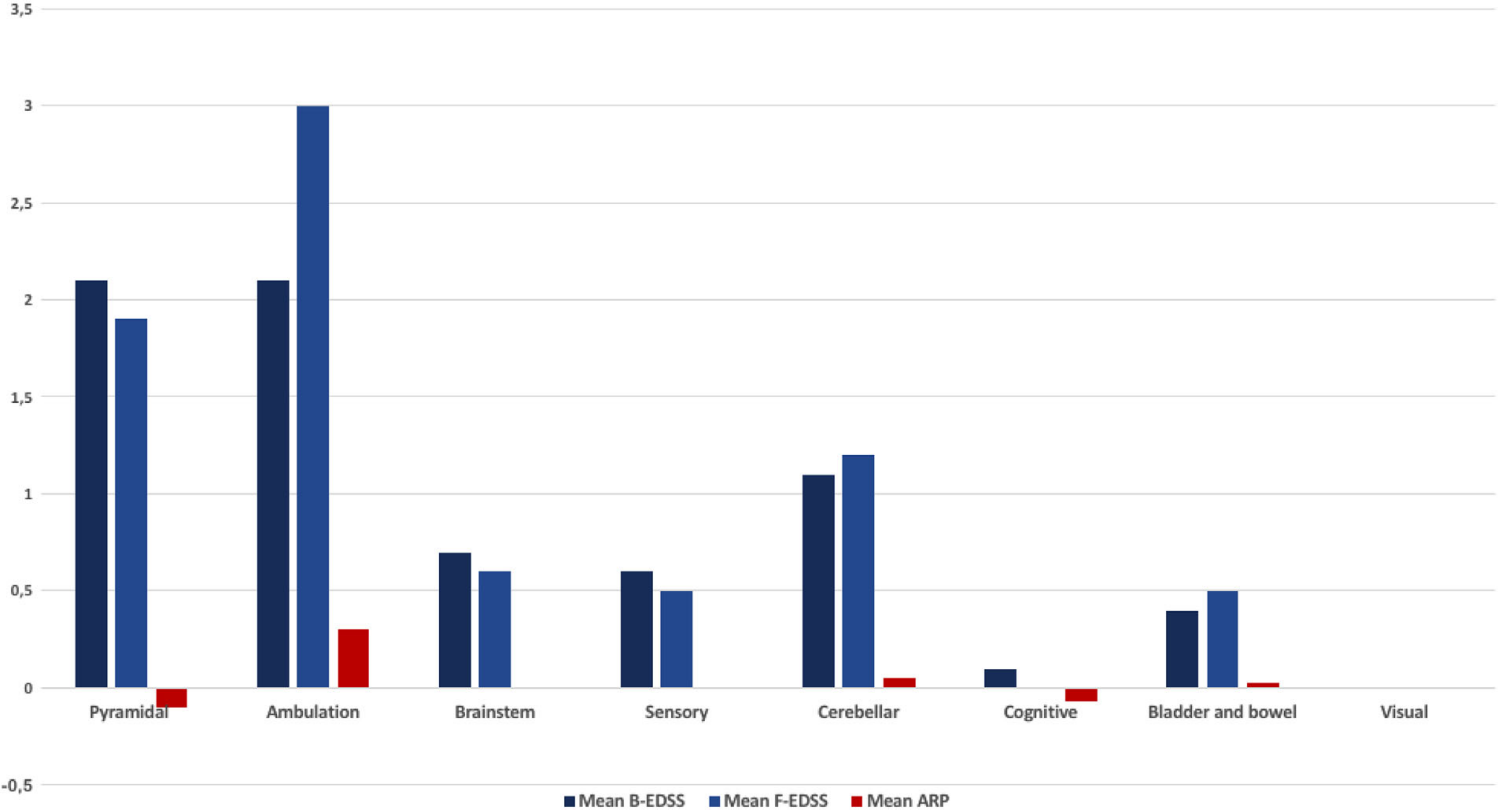
Function‐based mean B‐EDSS and F‐EDSS scores at baseline and final timepoints along with their corresponding annual progression rates *Abbreviation*: ARP, annual rate of progression

As for the B‐EDSS, the highest scores were noted in ambulation (2.1±2.6 points), pyramidal function (2±1 points), and cerebellar function (1.13±1.6 points) (Figure 2).

### 3.3 Clinical progression of the disability

After initiation of treatment, the ARR decreased from 1.6 ± 1 relapse/year before the initiation of NTZ to 0.2 ±1 relapse/year after switching to NTZ (p=0.325).

The mean F‐EDSS score was of 3.8 ± 2.26 points [1, 6.5] which was higher than the mean B‐EDSS. Yet, the difference remains statistically insignificant (p=0.954). Increase in the ambulation score (p=0.000,r=−0.834) and the cerebellar function (p=0.000,r=0.911) were the main significant variations (Figure 2).

The mean ARP of EDSS score was of 0.13 points/year. ARP of the different functions is represented in Figure 2. The APR of the cerebellar function was correlated with the AAR of the GM of the cerebellum during the first 2 years of NTZ treatment (p=0.033,r=−0.909).

### 3.4 Cognitive and psychiatric assessment

The main findings of the different cognitive tests and questionnaires are summarized in Table 2.

**TABLE 2:** Results of the cognitive and psychiatric evaluation in our patients

Test/questionnaire	Results
QPC (mean ± SD; [min; max]) Altered QPC (*n*)	5.4 ± 3 [0; 10] 16
Verbal fluency (mean ± SD) altered verbal fluency (*n*)	9 ± 2.1 13
Phonemic fluency (mean number of words/min± SD) altered phonemic fluency (*n*)	6 ± 3 17
SDMT (mean ± SD; [mix; max])	20.8 ± 10 [0; 36]
SPART: immediate recall (mean ± SD; [mix; max]) SPART: delayed recall (mean score ± SD)	14 ± 4 [6; 22] 4 ± 1.6

The SDMT score correlated with the cerebellar function at baseline (p=0.028,r=−0.506) and during the final assessment (p=0.002,r=−0.789). As for the SPART score, it was correlated with the baseline thalamic volume (p=0.008,r=−0.849).

### 3.5 MRI volumetric findings and assessment of atrophy and its clinical correlates

**FIGURE 3:**
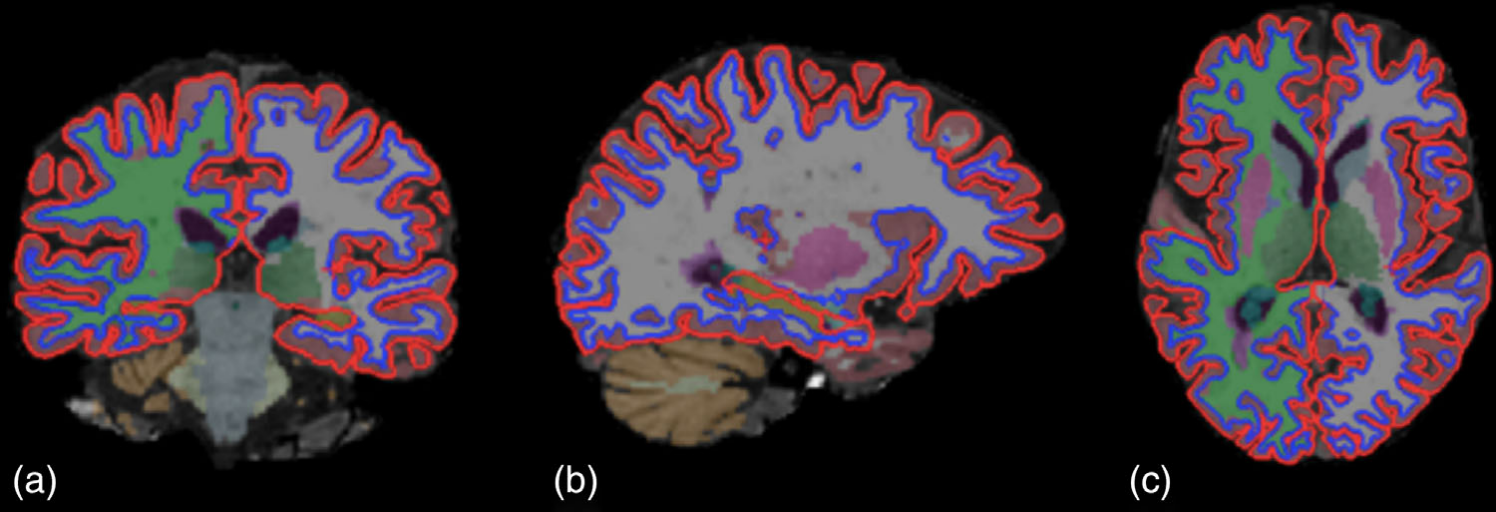
Results of FreeSurfer automated segmentation pipeline of T1 MRI into different anatomical brain regions in (a) the coronal plane, (b) sagittal plane, and (c) axial plane

Different volumes of segmented structures (Figure 3) were compared at two different time intervals:

– *During the first 2 years of treatment (T1–T0)*

**FIGURE 4:**
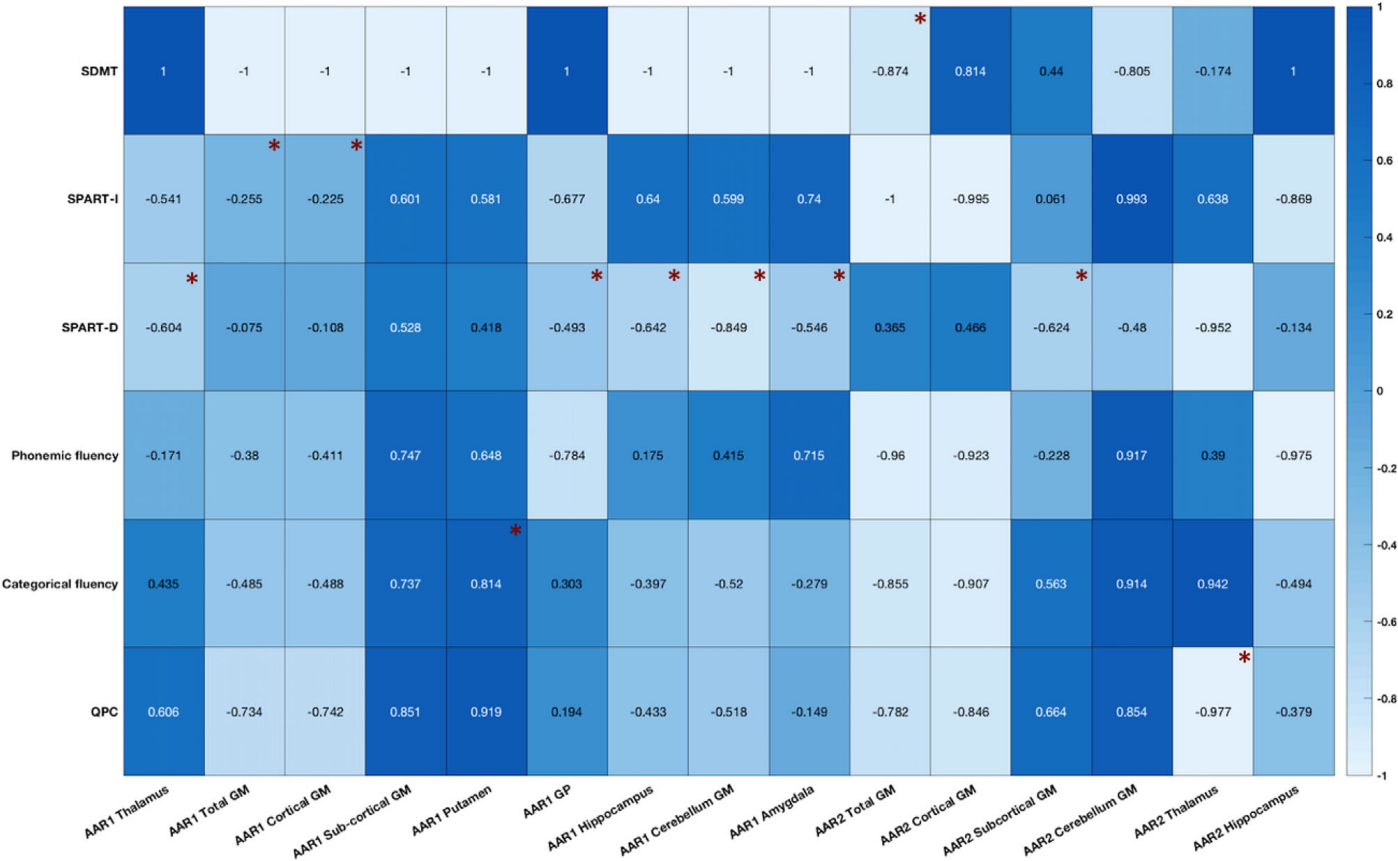
Correlations (Pearson's coefficient “*r*”) between the AAR of different brain regions and cognitive assessment findings. The “*” indicates a *p* value < .05. *Abbreviations*: *AAR1*, AAR during the first two years of NTZ therapy; AAR2, AAR during the third and fourth years of treatment; *SPART‐D*, SPART delayed recall; *SPART‐I*, SPART immediate recall

As detailed in Table 3, the volume of corpus callosum decreased significantly (p=0.032) along with the cerebellum GM volume (p=0.047). The AAR of the thalamus, total GM, cortical GM, GP, and the hippocampus correlated with SPART test score (Figure 4). As for the ARP of the cerebellar function, it was negatively correlated with AAR of the cerebellum GM (p=0.033,r=−0,909).

– *During the third and fourth years of treatment (T2–T1)*

**TABLE 3:** Comparison of results of MRI volumetric findings and AAR from baseline (T0) to T1 and from T1 to T2 timepoints

Structures	Mean volume (mm^3^) at baseline (T0)	Mean volume (mm^3^) at T1	*p* (comparison of volumes at T0 and T1)	Mean volume (mm^3^) at T2	*p* (comparison of volumes at T1 and T2)	AAR (T1–T0) (mm^3^/year)	AAR (T2–T1) (mm^3^/year)	*p*
IC	1,129,399.25	1,034,891.37	.071	1,045,176	.122	−58,741	43,941	.561
Total GM	457,275.25	482,317.25	.245	402,829.33	**.038**	55,733	−35,892	.669
Cortical GM	30,7577.62	336,390.50	.259	262,836	**.005**	53,627	−38,034	.568
Subcortical GM	47,981.12	45,263	.802	45,712	.336	199	2143	.912
Thalamus	13,131.25	12,346	.748	13,034	**.016**	904	1089	.464
Putamen	7786	6615.37	.213	6003	.077	91	−114	.799
Globus pallidus	2191.12	2099.25	.281	2056	.529	−113	146	.054
Caudate nucleus	4747	4824.25	.365	4324.33	.158	407	−203	**.008**
Amygdala	2461.87	4071.12	.05	2674	.252	1316	−3811	**.003**
Total WM	643,887.62	524,959.12	.73	613,421	.06	−113,637	77,459	.697
Corpus callosum	2179.62	1664.87	**0.032**	1363	.124	1316	168	.112
Cerebellum (total)	131,619.37	128,247.12	.273	124,114	.564	21	3285	.953
WM cerebellum	30,479.87	28,086.62	.416	29,830.66	.333	−1696	2735	.074
GM cerebellum	101,139.50	100,160.5	**.047**	94,283.33	.465	1718	549	.77
Hippocampus	6836	6980.87	.345	8040	.914	718	809	.366

In bold are the p values which are statistically significant, meaning a p value < 0.05.

Regarding the GM, significant atrophy was noted (p=0.038) mainly due to cortical atrophy (p=0.005). As for the subcortical GM, it was globally stable. It is noteworthy that the thalamus volume increased significantly (p=0.016).

The AAR of the cortical GM and the cerebellum GM during the third and fourth years of treatment were negatively correlated with the QPC score. Categorical fluency score was positively correlated with the AAR (T2–T1) of the putamen and the thalamus (Figure 4).

While comparing the AAR of the two first years of treatment and the third and fourth years, we noted that the CN and the amygdala were subject to significant progression of annual atrophy rate (Table 3).

## 4 DISCUSSION

In our study, RR‐MS patients treated with NTZ showed a regional atrophy pattern that progressed dynamically during the 4‐year treatment period. The two first years of treatment mainly reflected a reduction in the CC volume and the cerebellum GM. The third and the fourth years of treatment were characterized with the reduction in GM volume mainly the cortical one. As for the cognitive profile, memory impairment assed using the 10/36 SPART test correlated with the AAR of the cortical GM, subcortical GM including the thalamus and the GP during the first two years of treatment. It was also associated to the AAR of the hippocampus and the amygdala.

### 4.1 The atrophy pattern: A dynamic feature

In accordance with previous studies (Andravizou et al., 2019; Arpín et al., 2016; Sastre‐Garriga et al., 2015; Talmage et al., 2017; Vidal‐Jordana et al., 2013; Zivadinov et al., 2008), early WM loss is a hallmark of the atrophy generated during the first two years of NTZ treatment. Our study demonstrated a significant reduction in the volume of the CC. Such wide thick WM tract might be considered as a valuable marker of neurodegeneration in MS (Figueir et al., 2007; Yaldizli et al., 2010). Representing a region of predilection of MS lesions, the atrophy of CC is mainly due to the anti‐inflammatory activity of the treatment, leading to the regression of the vasogenic edema within the demyelination plaques and causing a pseudoatrophy effect.

However, the pseudo‐atrophy seems to be an overlapping phenomenon unrestricted to white matter. In fact, demyelination is seen in the cerebellar GM five times more than in the white matter (Gilmore et al., 2009). Meningeal inflammation in the deep folia accommodates a persistent inflammation in direct contact with the cortical GM of the cerebellum (Howell et al., 2011), which explains the decrease in its GM volume due to the anti‐inflammatory effect of the treatment as demonstrated in our study.

Regarding supra‐tentorial GM, our study demonstrated a significant loss in the cortical GM volume (p=0.005) and, as a consequence, the total GM (p=0.038) during the third and fourth years of treatment. At first glance, such finding might be considered atypical since NTZ is known to slowdown the atrophy of GM beyond the two first years of treatment once the pseudoatrophy phenomenon had taken place (Andravizou et al., 2019; Sastre‐Garriga et al., 2015). Based on the recent studies (Calabrese et al., 2015; Ciampi et al., 2017; De Stefano et al., 2021; Eshaghi et al., 2018; Sotirchos et al., 2020), it may be postulated that GM is the main target of neurodegeneration regardless of the disease stability, conducted treatment and regression of active inflammatory lesions. Pseudoatrophy might be a valid hypothesis for GM loss expanded beyond the first 2 years of treatment. Yet, data on pseudoatrophy with the GM remain discordant, making the interpretation of GM reduction even harder to fully encapsulate (De Stefano et al., 2021). It may be attributed to the real ongoing neurodegenerative process based on several mechanisms such as mitochondrial failure, iron deposition, retrograde degeneration through white matter lesions, and sustained meningeal inflammation (Calabrese et al., 2015; Preziosa, Rocca, Riccitelli, et al., 2020).

As for subcortical GM, it remained globally stable with a significant increase in the thalamus volume which may indicate a neuroprotective effect of NTZ by acting on specific regions of brain that are highly implicated in the cognitive aspect in MS such as the thalamus (Bisecco et al., 2021; DeLuca et al., 2015; Rojas et al., 2018; Schoonheim et al., 2015).

Regarding the dynamic aspect of atrophy, the CN showed a progression of the AAR (p=0.008). Such fact may not be specific to MS patients treated with NTZ since it has already been established as an early characteristic marker of atrophy in the relapsing remitted form of the disease (Eshaghi et al., 2018). Such finding might be comprehensible considering that, on a histopathological level, the CN is one of the most affected deep GM regions by the demyelination process (Haider et al., 2014).

### 4.2 Cognitive mapping of MS patients treated with NTZ

Exploring the relationship between the volumes and AAR of different brain regions and the cognitive profile of MS patients helped addressing further the highly cognitive regions in MS patients treated with NTZ.

As a matter of fact, 16 of our patients expressed considerable cognitive complaint. The QPC score was correlated with the volume of the cerebellum GM at baseline and the AAR of the cerebellum GM during the third and fourth years of treatment (Figure 4). Such link could be explained by the fact that the cerebellum pathology in MS is greatly determinative of both physical disability and cognitive decline (Damasceno et al., 2014). Cognitive dysfunction may be the result of the disruption of the cerebello‐cortical loops established with the prefrontal cortex and the lateral parietal cortex via the thalamus and the pons (Habas et al., 2009; Krienen & Buckner, 2009). The implication of the disruption of the same neuronal network encompasses the correlation of the SDMT score with the cerebellar function at baseline (p=0.028) and during the final assessment (p=0.002). In line with our findings, Cerasa et al. (2012) distinguished MS patients with cerebellar clinical signs as patients with a more considerable cognitive dysfunction compared to MS patients with no clinical cerebellar signs.

Regarding memory evaluation, our study highlighted that the AAR of GM, cortical GM, and specific regions of the subcortical GM along with the hippocampus were associated to SPART score (Figure 4). In fact, cortical GM includes the orbitofrontal cortex and the medial prefrontal cortex, which are functionally linked to the hippocampus via a network for memory formation, maintenance, and retrieval (Benarroch, 2013). Furthermore, the SPART score was also correlated with the baseline thalamic volume and with the AAR of the thalamus. Such finding lines with the currently sparse literature pinpointing the cognitive function of the thalamus in MS including specific areas such as the ventral anterior nucleus and ventral lateral nuclei (Bisecco et al., 2015, 2021). Visuospatial memory seems to be altered in thalamus pathology, and it is explained partly by the disruption of the thalamo‐frontal circuits and the connection between the medio‐dorsal thalamus and the hippocampus (Parnaudeau et al., 2018). However, even though the AAR of the thalamus correlated with the SPART score, the fact that the thalamus volume increased significantly during the third and fourth years of treatment and that baseline volume was already associated to a greater SPART score could reflect that NTZ may have decelerated neurodegeneration within the thalamus. Thus, memory impairment might be just a sequela of the damage prior to the initiation of NTZ therapy or might be due to the neurodegenerative process of the disease going at its normal pace.

Our results also showed that SPART delayed recall test was correlated with the AAR of the amygdala (Figure 4), replicating the finding that the amygdala is linked to the visuospatial memory and psychosocial functioning in pediatric onset MS patients (Green et al., 2018). The increasing AAR of the amygdala, as shown in our study (p=0.003), calls for a greater attention to this structure as it may particularly be vulnerable to the neurodegenerative process with great cognitive, emotional, and social impact.

As for the GP, its AAR correlated, as well, with the SPART score (Figure 4) which is plausible given the intricate involvement of deep GM in memory. In fact, the GP is implicated in memory networks involving subsets of cortico‐basal loops (Middleton & Strick, 2000).

### 4.3 Physical disability and the neurodegenerative process

The progression of the cerebellar signs was the key clinical feature that correlated with volumetric measures. It was correlated with the AAR of the GM of the cerebellum during the first year of treatment. Such finding emphasizes further the crucial role of the cerebellum in causing both physical and cognitive handicap in MS (Damasceno et al., 2014; Weier et al., 2014).

However, our study is not without limitations. The seemingly small sample size might be explained by the fact that Tunisia is characterized with a moderate prevalence of MS ranging from 9 to 20 per 100,000 habitants and by the adopted exclusion criteria (Yamout et al., 2020). Even though the sample size may not allow to draw definite conclusion about the impact of NTZ on atrophy progression, it provides preliminary data that calls for further investigations. The single timepoint assessment of the cognitive profile did not allow to better study the cognitive aspect and its progression since the initiation of NTZ.

## 5 CONCLUSION

Through this study, we analyzed a large number of clinical, neurocognitive, and MRI variables acquired at baseline and during the first 4 years of NTZ treatment. A robust and consistent finding is that atrophy affects white matter during the first 2 years of treatment and the GM later. A key finding to highlight is the increase in the thalamus volume, which may indicate a neuroprotective effect of NTZ. This study also allowed to map vulnerable highly cognitive regions involved in memory and executive function such as the cortical GM, the cerebellar GM, the deep GM mainly the thalamus, the GT, and the putamen. It emphasized the particular vulnerability of the caudate nucleus to neurodegeneration with an increasing atrophy rate during the third and fourth years of treatment.

This study also opens the door for future research that could conduct long‐term follow‐up of patients before and after the initiation of NTZ with larger samples and appropriate controls in order to better comprehend the supplemental impact of NTZ on atrophy progression. By highlighting brain regions highly involved in cognition, our study provides insight into the potential role of these structures and the possibility to use them as biomarkers of the disease progression.

## CONFLICT OF INTEREST

The authors declare no conflict of interest.

### PEER REVIEW

The peer review history for this article is available at https://publons.com/publon/10.1002/brb3.2573

## Data Availability

The data that support the findings of this study are available on request from the corresponding author. The data are not publicly available due to privacy or ethical restrictions.

## References

[brb32573-bib-0001] Alvarez, E. , Nair, K. V. , Hoyt, B. D. , Seale, R. A. , Sillau, S. , Miravalle, A. , Engebretson, E. , Schurr, B. , Corboy, J. R. , Vollmer, T. L. , & Honce, J. M. (2021). Brain atrophy rates in patients with multiple sclerosis on long term natalizumab resembles healthy controls. Multiple Sclerosis and Related Disorders, 55, 103170. 10.1016/j.msard.2021.103170 34364034

[brb32573-bib-0002] Andravizou, A. , Dardiotis, E. , Artemiadis, A. , Sokratous, M. , Siokas, V. , Tsouris, Z. , Aloizou, A.‐M. , Nikolaidis, I. , Bakirtzis, C. , Tsivgoulis, G. , Deretzi, G. , Grigoriadis, N. , Bogdanos, D. P. , & Hadjigeorgiou, G. M. (2019). Brain atrophy in multiple sclerosis: Mechanisms, clinical relevance and treatment options. Autoimmunity Highlights, 10(1), 7. 10.1186/s13317-019-0117-5 32257063 PMC7065319

[brb32573-bib-0003] Arpín, E. C. , Sobrino, T. G. , Vivero, C. D. , del Campo Amigo Jorrín, M. , Regal, A. R. , González, J. P. , & Bouzas, M. L. (2016). Changes in brain atrophy indices in patients with relapsing‐remitting multiple sclerosis treated with natalizumab. Neurodegenerative Disease Management, 6(1), 5–12. 10.2217/nmt.15.53 26782312

[brb32573-bib-0004] Barois, E. , Sagawa, Y. , Yilmaz, S. , Magnin, E. , & Decavel, P. (2020). What (more) can verbal fluency tell us about multiple sclerosis? Annals of Physical and Rehabilitation Medicine. Advance online publication. 10.1016/j.rehab.2020.05.002 32450272

[brb32573-bib-0005] Benarroch, E. E. (2013). Adult neurogenesis in the dentate gyrus: General concepts and potential implications. Neurology, 81(16), 1443–1452. 10.1212/WNL.0b013e3182a9a156 24078736

[brb32573-bib-0006] Benedict, R. H. , DeLuca, J. , Phillips, G. , LaRocca, N. , Hudson, L. D. , & Rudick, R. , & Multiple Sclerosis Outcome Assessments Consortium . (2017). Validity of the symbol digit modalities test as a cognition performance outcome measure for multiple sclerosis. Multiple Sclerosis (Houndmills, Basingstoke, England), 23(5), 721–733. 10.1177/1352458517690821 PMC5405816 28206827

[brb32573-bib-0007] Bisecco, A. , Capuano, R. , Caiazzo, G. , d'Ambrosio, A. , Docimo, R. , Cirillo, M. , Russo, A. , Altieri, M. , Bonavita, S. , Rocca, M. A. , Filippi, M. , Tedeschi, G. , & Gallo, A. (2021). Regional changes in thalamic shape and volume are related to cognitive performance in multiple sclerosis. Multiple Sclerosis (Houndmills, Basingstoke, England), 27(1), 134–138. 10.1177/1352458519892552 31793399

[brb32573-bib-0008] Bisecco, A. , Rocca, M. A. , Pagani, E. , Mancini, L. , Enzinger, C. , Gallo, A. , Vrenken, H. , Stromillo, M. L. , Copetti, M. , Thomas, D. L. , Fazekas, F. , Tedeschi, G. , Barkhof, F. , Stefano, N. D. , & Filippi, M. ; MAGNIMS Network . (2015). Connectivity‐based parcellation of the thalamus in multiple sclerosis and its implications for cognitive impairment: A multicenter study. Human Brain Mapping, 36(7), 2809–2825. 10.1002/hbm.22809 25873194 PMC6869750

[brb32573-bib-0009] Calabrese, M. , Magliozzi, R. , Ciccarelli, O. , Geurts, J. J. G. , Reynolds, R. , & Martin, R. (2015). Exploring the origins of grey matter damage in multiple sclerosis. Nature Reviews. Neuroscience, 16(3), 147–158. 10.1038/nrn3900 25697158

[brb32573-bib-0010] Cerasa, A. , Passamonti, L. , Valentino, P. , Nisticò, R. , Pirritano, D. , Gioia, M. C. , Chiriaco, C. , Mangone, G. , Perrotta, P. , & Quattrone, A. (2012). Cerebellar‐parietal dysfunctions in multiple sclerosis patients with cerebellar signs. Experimental Neurology, 237(2), 418–426. 10.1016/j.expneurol.2012.07.020 22892245

[brb32573-bib-0011] Ciampi, E. , Pareto, D. , Sastre‐Garriga, J. , Vidal‐Jordana, A. , Tur, C. , Río, J. , Tintoré, M. , Auger, C. , Rovira, A. , & Montalban, X. (2017). Grey matter atrophy is associated with disability increase in natalizumab‐treated patients. Multiple Sclerosis (Houndmills, Basingstoke, England), 23(4), 556–566. 10.1177/1352458516656808 27354019

[brb32573-bib-0012] Collins, D. L. , Neelin, P. , Peters, T. M. , & Evans, A. C. (1994). Automatic 3D intersubject registration of MR volumetric data in standardized Talairach space. Journal of Computer Assisted Tomography, 18(2), 192–205. 8126267

[brb32573-bib-0013] Compston, A. , & Coles, A. (2002). Multiple sclerosis. The Lancet, 359(9313), 1221–1231. 10.1016/S0140-6736(02)08220-X 11955556

[brb32573-bib-0014] Damasceno, A. , Damasceno, B. P. , & Cendes, F. (2014). The clinical impact of cerebellar grey matter pathology in multiple sclerosis. Plos One, 9(5), e96193. 10.1371/journal.pone.0096193 24789257 PMC4008536

[brb32573-bib-0015] De Stefano, N. , Giorgio, A. , Gentile, G. , Stromillo, M. L. , Cortese, R. , Gasperini, C. , Visconti, A. , Sormani, M. P. , & Battaglini, M. (2021). Dynamics of pseudo‐atrophy in RRMS reveals predominant gray matter compartmentalization. Annals of Clinical and Translational Neurology, 8(3), 623–630. 10.1002/acn3.51302 33534940 PMC7951094

[brb32573-bib-0016] Delgado‐Álvarez, A. , Matias‐Guiu, J. A. , Delgado‐Alonso, C. , Hernández‐Lorenzo, L. , Cortés‐Martínez, A. , Vidorreta, L. , Montero‐Escribano, P. , Pytel, V. , & Matias‐Guiu, J. (2021). Cognitive processes underlying verbal fluency in multiple sclerosis. Frontiers in Neurology, 11, 629183. 10.3389/fneur.2020.629183 33551984 PMC7859643

[brb32573-bib-0017] DeLuca, G. C. , Yates, R. L. , Beale, H. , & Morrow, S. A. (2015). Cognitive impairment in multiple sclerosis: Clinical, radiologic and pathologic insights. Brain Pathology (Zurich, Switzerland), 25(1), 79–98. 10.1111/bpa.12220 PMC8029470 25521179

[brb32573-bib-0018] Dent, A. , & Lincoln, N. B. (2000). Screening for memory problems in multiple sclerosis. The British Journal of Clinical Psychology, 39(3), 311–315. 10.1348/014466500163329 11033753

[brb32573-bib-0019] Eshaghi, A. , Marinescu, R. V. , Young, A. L. , Firth, N. C. , Prados, F. , Jorge Cardoso, M. , Tur, C. , De Angelis, F. , Cawley, N. , Brownlee, W. J. , De Stefano, N. , Laura Stromillo, M. , Battaglini, M. , Ruggieri, S. , Gasperini, C. , Filippi, M. , Rocca, M. A. , Rovira, A. , Sastre‐Garriga, J. , … Ciccarelli, O. (2018). Progression of regional grey matter atrophy in multiple sclerosis. Brain: A Journal of Neurology, 141(6), 1665–1677. 10.1093/brain/awy088 29741648 PMC5995197

[brb32573-bib-0020] Figueira, F. F. A. , Santos, V. S. d. , Figueira, G. M. A. , & Silva, A. C. M. d. (2007). Corpus callosum index: A practical method for long‐term follow‐up in multiple sclerosis. Arquivos De Neuro‐Psiquiatria, 65(4A), 931–935. 10.1590/s0004-282X2007000600001 18094848

[brb32573-bib-0021] Fischl, B. , Salat, D. H. , Busa, E. , Albert, M. , Dieterich, M. , Haselgrove, C. , van der Kouwe, A. , Killiany, R. , Kennedy, D. , Klaveness, S. , Montillo, A. , Makris, N. , Rosen, B. , & Dale, A. M. (2002). Whole brain segmentation: Automated labeling of neuroanatomical structures in the human brain. Neuron, 33(3), 341–355. 10.1016/s0896-6273(02)00569-x 11832223

[brb32573-bib-0022] Fischl, B. , van der Kouwe, A. , Destrieux, C. , Halgren, E. , Ségonne, F. , Salat, D. H. , Busa, E. , Seidman, L. J. , Goldstein, J. , Kennedy, D. , Caviness, V. , Makris, N. , Rosen, B. , & Dale, A. M. (2004). Automatically parcellating the human cerebral cortex. Cerebral Cortex (New York, N.Y.: 1991), 14(1), 11–22. 10.1093/cercor/bhg087 14654453

[brb32573-bib-0023] Gerstenecker, A. , Martin, R. , Marson, D. C. , Bashir, K. , & Triebel, K. L. (2016). Introducing demographic‐corrections for the 10/36 spatial recall test. International Journal of Geriatric Psychiatry, 31(4), 406–411. 10.1002/gps.4346 26270773 PMC4752917

[brb32573-bib-0024] Gilmore, C. P. , Donaldson, I. , Bö, L. , Owens, T. , Lowe, J. , & Evangelou, N. (2009). Regional variations in the extent and pattern of grey matter demyelination in multiple sclerosis: A comparison between the cerebral cortex, cerebellar cortex, deep grey matter nuclei and the spinal cord. Journal of Neurology, Neurosurgery, and Psychiatry, 80(2), 182–187. 10.1136/jnnp.2008.148767 18829630

[brb32573-bib-0025] Green, R. , Adler, A. , Banwell, B. L. , Fabri, T. L. , Yeh, E. A. , Collins, D. L. , Sled, J. G. , Narayanan, S. , & Till, C. (2018). Involvement of the amygdala in memory and psychosocial functioning in pediatric‐onset multiple sclerosis. Developmental Neuropsychology, 43(6), 524–534. 10.1080/87565641.2018.1485679 29911891

[brb32573-bib-0026] Habas, C. , Kamdar, N. , Nguyen, D. , Prater, K. , Beckmann, C. F. , Menon, V. , & Greicius, M. D. (2009). Distinct cerebellar contributions to intrinsic connectivity networks. The Journal of Neuroscience: The Official Journal of the Society for Neuroscience, 29(26), 8586–8594. 10.1523/JNEUROSCI.1868-09.2009 19571149 PMC2742620

[brb32573-bib-0027] Haider, L. , Simeonidou, C. , Steinberger, G. , Hametner, S. , Grigoriadis, N. , Deretzi, G. , Kovacs, G. G. , Kutzelnigg, A. , Lassmann, H. , & Frischer, J. M. (2014). Multiple sclerosis deep grey matter: The relation between demyelination, neurodegeneration, inflammation and iron. Journal of Neurology, Neurosurgery, and Psychiatry, 85(12), 1386–1395. 10.1136/jnnp-2014-307712 24899728 PMC4251183

[brb32573-bib-0028] Howell, O. W. , Reeves, C. A. , Nicholas, R. , Carassiti, D. , Radotra, B. , Gentleman, S. M. , Serafini, B. , Aloisi, F. , Roncaroli, F. , Magliozzi, R. , & Reynolds, R. (2011). Meningeal inflammation is widespread and linked to cortical pathology in multiple sclerosis. Brain, 134(9), 2755–2771. 10.1093/brain/awr182 21840891

[brb32573-bib-0029] Kalb, R. , Beier, M. , Benedict, R. H. , Charvet, L. , Costello, K. , Feinstein, A. , Gingold, J. , Goverover, Y. , Halper, J. , Harris, C. , Kostich, L. , Krupp, L. , Lathi, E. , LaRocca, N. , Thrower, B. , & DeLuca, J. (2018). Recommendations for cognitive screening and management in multiple sclerosis care. Multiple Sclerosis, 24(13), 1665–1680. 10.1177/1352458518803785 30303036 PMC6238181

[brb32573-bib-0030] Kotelnikova, E. , Kiani, N. A. , Abad, E. , Martinez‐Lapiscina, E. H. , Andorra, M. , Zubizarreta, I. , Pulido‐Valdeolivas, I. , Pertsovskaya, I. , Alexopoulos, L. G. , Olsson, T. , Martin, R. , Paul, F. , Tegnér, J. , Garcia‐Ojalvo, J. , & Villoslada, P. (2017). Dynamics and heterogeneity of brain damage in multiple sclerosis. PLoS Computational Biology, 13(10), e1005757. 10.1371/journal.pcbi.1005757 29073203 PMC5657613

[brb32573-bib-0031] Krienen, F. M. , & Buckner, R. L. (2009). Segregated fronto‐cerebellar circuits revealed by intrinsic functional connectivity. Cerebral Cortex, 19(10), 2485–2497. 10.1093/cercor/bhp135 19592571 PMC2742600

[brb32573-bib-0032] Lublin, F. D. , Reingold, S. C. , Cohen, J. A. , Cutter, G. R. , Sørensen, P. S. , Thompson, A. J. , Wolinsky, J. S. , Balcer, L. J. , Banwell, B. , Barkhof, F. , Bebo, B. , Calabresi, P. A. , Clanet, M. , Comi, G. , Fox, R. J. , Freedman, M. S. , Goodman, A. D. , Inglese, M. , Kappos, L. , & Polman, C. H. (2014). Defining the clinical course of multiple sclerosis. Neurology, 83(3), 278–286. 10.1212/WNL.0000000000000560 24871874 PMC4117366

[brb32573-bib-0033] Masson, E. (n.d.). Le Questionnaire de Plainte Cognitive (QPC): Outil de dépistage de la plainte des sujets présentant une maladie d'Alzheimer ou un MCI. EM‐Consulte. https://www.em‐consulte.com/article/104972/le‐questionnaire‐de‐plainte‐cognitive‐qpc‐outil‐de

[brb32573-bib-0034] Menascu, S. , Stern, M. , Aloni, R. , Kalron, A. , Magalshvili, D. , & Achiron, A. (2019). Assessing cognitive performance in radiologically isolated syndrome. Multiple Sclerosis and Related Disorders, 32, 70–73. 10.1016/j.msard.2019.04.030 31054500

[brb32573-bib-0035] Middleton, F. A. , & Strick, P. L. (2000). Basal ganglia output and cognition: Evidence from anatomical, behavioral, and clinical studies. Brain and Cognition, 42(2), 183–200. 10.1006/brcg.1999.1099 10744919

[brb32573-bib-0036] Oset, M. , Stasiolek, M. , & Matysiak, M. (2020). Cognitive dysfunction in the early stages of multiple sclerosis—How much and how important? Current Neurology and Neuroscience Reports, 20(7), 22. 10.1007/s11910-020-01045-3 32444997 PMC7244611

[brb32573-bib-0037] Parnaudeau, S. , Bolkan, S. S. , & Kellendonk, C. (2018). The mediodorsal thalamus: An essential partner of the prefrontal cortex for cognition. Biological Psychiatry, 83(8), 648–656. 10.1016/j.biopsych.2017.11.008 29275841 PMC5862748

[brb32573-bib-0038] Polman, C. H. , O'Connor, P. W. , Havrdova, E. , Hutchinson, M. , Kappos, L. , Miller, D. H. , Phillips, J. T. , Lublin, F. D. , Giovannoni, G. , Wajgt, A. , Toal, M. , Lynn, F. , Panzara, M. A. , & Sandrock, A. W. ; AFFIRM Investigators . (2006). A randomized, placebo‐controlled trial of natalizumab for relapsing multiple sclerosis. The New England Journal of Medicine, 354(9), 899–910. 10.1056/NEJMoa044397 16510744

[brb32573-bib-0039] Preziosa, P. , Rocca, M. A. , Pagani, E. , Storelli, L. , Rodegher, M. , Moiola, L. , & Filippi, M. (2020). Two‐year regional grey and white matter volume changes with natalizumab and fingolimod. Journal of Neurology, Neurosurgery, and Psychiatry, 91(5), 493–502. 10.1136/jnnp-2019-322439 32111638

[brb32573-bib-0040] Preziosa, P. , Rocca, M. A. , Riccitelli, G. C. , Moiola, L. , Storelli, L. , Rodegher, M. , Comi, G. , Signori, A. , Falini, A. , & Filippi, M. (2020). Effects of natalizumab and fingolimod on clinical, cognitive, and magnetic resonance imaging measures in multiple sclerosis. Neurotherapeutics: The Journal of the American Society for Experimental NeuroTherapeutics, 17(1), 208–217. 10.1007/s13311-019-00781-w 31452082 PMC7007466

[brb32573-bib-0041] Prins, M. , Schul, E. , Geurts, J. , van der Valk, P. , Drukarch, B. , & van Dam, A.‐M. (2015). Pathological differences between white and grey matter multiple sclerosis lesions. Annals of the New York Academy of Sciences, 1351, 99–113. 10.1111/nyas.12841 26200258

[brb32573-bib-0042] Rojas, J. I. , Murphy, G. , Sanchez, F. , Patrucco, L. , Fernandez, M. C. , Miguez, J. , Funes, J. , Golimstok, A. , & Cristiano, E. (2018). Thalamus volume change and cognitive impairment in early relapsing‐remitting multiple sclerosis patients. The Neuroradiology Journal, 31(4), 350–355. 10.1177/1971400918781977 29869576 PMC6111418

[brb32573-bib-0043] Sastre‐Garriga, J. , Tur, C. , Pareto, D. , Vidal‐Jordana, A. , Auger, C. , Río, J. , Huerga, E. , Tintoré, M. , Rovira, A. , & Montalban, X. (2015). Brain atrophy in natalizumab‐treated patients: A 3‐year follow‐up. Multiple Sclerosis (Houndmills, Basingstoke, England), 21(6), 749–756. 10.1177/1352458514556300 25392330

[brb32573-bib-0044] Schoonheim, M. M. , Hulst, H. E. , Brandt, R. B. , Strik, M. , Wink, A. M. , Uitdehaag, B. M. J. , Barkhof, F. , & Geurts, J. J. G. (2015). Thalamus structure and function determine severity of cognitive impairment in multiple sclerosis. Neurology, 84(8), 776–783. 10.1212/WNL.0000000000001285 25616483

[brb32573-bib-0045] Ségonne, F. , Dale, A. M. , Busa, E. , Glessner, M. , Salat, D. , Hahn, H. K. , & Fischl, B. (2004). A hybrid approach to the skull stripping problem in MRI. Neuroimage, 22(3), 1060–1075. 10.1016/j.neuroimage.2004.03.032 15219578

[brb32573-bib-0046] Sotirchos, E. S. , Gonzalez‐Caldito, N. , Dewey, B. E. , Fitzgerald, K. C. , Glaister, J. , Filippatou, A. , Ogbuokiri, E. , Feldman, S. , Kwakyi, O. , Risher, H. , Crainiceanu, C. , Pham, D. L. , Van Zijl, P. C. , Mowry, E. M. , Reich, D. S. , Prince, J. L. , Calabresi, P. A. , & Saidha, S. (2020). Effect of disease‐modifying therapies on subcortical gray matter atrophy in multiple sclerosis. Multiple Sclerosis (Houndmills, Basingstoke, England), 26(3), 312–321. 10.1177/1352458519826364 PMC6689465 30741108

[brb32573-bib-0047] Talmage, G. D. , Coppes, O. J. M. , Javed, A. , & Bernard, J. (2017). Natalizumab stabilizes physical, cognitive, MRI, and OCT markers of disease activity: A prospective, non‐randomized pilot study. Plos One, 12(4), e0173299. 10.1371/journal.pone.0173299 28426702 PMC5398512

[brb32573-bib-0048] Thompson, A. J. , Banwell, B. L. , Barkhof, F. , Carroll, W. M. , Coetzee, T. , Comi, G. , Correale, J. , Fazekas, F. , Filippi, M. , Freedman, M. S. , Fujihara, K. , Galetta, S. L. , Hartung, H. P. , Kappos, L. , Lublin, F. D. , Marrie, R. A. , Miller, A. E. , Miller, D. H. , Montalban, X. , … Cohen, J. A. (2018). Diagnosis of multiple sclerosis: 2017 revisions of the McDonald criteria. The Lancet Neurology, 17(2), 162–173. 10.1016/S1474-4422(17)30470-2 29275977

[brb32573-bib-0049] Vidal‐Jordana, A. , Sastre‐Garriga, J. , Pérez‐Miralles, F. , Tur, C. , Tintoré, M. , Horga, A. , Auger, C. , Río, J. , Nos, C. , Edo, M. C. , Arévalo, M. J. , Castilló, J. , Rovira, A. , & Montalban, X. (2013). Early brain pseudoatrophy while on natalizumab therapy is due to white matter volume changes. Multiple Sclerosis (Houndmills, Basingstoke, England), 19(9), 1175–1181. 10.1177/1352458512473190 23319072

[brb32573-bib-0050] Weier, K. , Penner, I. K. , Magon, S. , Amann, M. , Naegelin, Y. , Andelova, M. , Derfuss, T. , Stippich, C. , Radue, E.‐W. , Kappos, L. , & Sprenger, T. (2014). Cerebellar abnormalities contribute to disability including cognitive impairment in multiple sclerosis. Plos One, 9(1), e86916. 10.1371/journal.pone.0086916 24466290 PMC3899307

[brb32573-bib-0051] Yaakub, S. N. , Heckemann, R. A. , Keller, S. S. , McGinnity, C. J. , Weber, B. , & Hammers, A. (2020). On brain atlas choice and automatic segmentation methods: A comparison of MAPER & FreeSurfer using three atlas databases. Scientific Reports, 10(1), 2837. 10.1038/s41598-020-57951-6 32071355 PMC7028906

[brb32573-bib-0052] Yaldizli, O. , Atefy, R. , Gass, A. , Sturm, D. , Glassl, S. , Tettenborn, B. , & Putzki, N. (2010). Corpus callosum index and long‐term disability in multiple sclerosis patients. Journal of Neurology, 257(8), 1256–1264. 10.1007/s00415-010-5503-x 20198382

[brb32573-bib-0053] Yamout, B. I. , Assaad, W. , Tamim, H. , Mrabet, S. , & Goueider, R. (2020). Epidemiology and phenotypes of multiple sclerosis in the Middle East North Africa (MENA) region. Multiple Sclerosis Journal – Experimental, Translational and Clinical, 6(1), 2055217319841881. 10.1177/2055217319841881 31984137 PMC6961141

[brb32573-bib-0054] Zivadinov, R. , Reder, A. T. , Filippi, M. , Minagar, A. , Stüve, O. , Lassmann, H. , Racke, M. K. , Dwyer, M. G. , Frohman, E. M. , & Khan, O. (2008). Mechanisms of action of disease‐modifying agents and brain volume changes in multiple sclerosis. Neurology, 71(2), 136–144. 10.1212/01.wnl.0000316810.01120.05 18606968

